# Women and Leadership of Cardiology and Oncology Clinical Trials—Swimming Against the Tide

**DOI:** 10.1001/jamanetworkopen.2022.0050

**Published:** 2022-02-01

**Authors:** Emily E. Moin, Nosheen Reza

**Affiliations:** Department of Medicine, Massachusetts General Hospital, Boston; Division of Cardiovascular Medicine, Department of Medicine, Perelman School of Medicine at the University of Pennsylvania, Philadelphia

Women are underrepresented in medicine across numerous specialties, both as authors of scholarly work and in positions of leadership.^1^ Growing interest in sex and gender representation in medicine has led to deeper scrutiny of these disparities, demonstrating the near-universality of the dearth of women in these roles and the lack of change in recent years. This inequity extends to clinical trial leadership, which is concerning, as clinical trials play a critical role in generating high-quality evidence to guide clinical practice. The benefits of increasing diversity in clinical trial leadership extend broadly to professionals and patients.^2^ Perhaps most importantly, multiple investigations have demonstrated that clinical trials with women lead authors enroll greater proportions of women participants.^2-4^ Thus, achieving commensurate representation of women in clinical trial leadership is a goal with both ideological and practical underpinnings: we seek to achieve equity in medicine on its own merits, as well as for its potential to propagate further benefits throughout society.

Elsewhere in *JAMA Network Open,* Shahid et al^5^ and Muquith et al^6^ present the results of work examining the representation of women investigators in clinical trials in cardiology and oncology, respectively. They not only redemonstrate the persistence of underrepresentation of women in each field but also identify deficiencies and opportunities for improvement that may have eluded prior, more general analyses.

Shahid et al^5^ narrow the focus of previous investigations of authorship of cardiovascular disease clinical trials to only pivotal trials of novel cardiovascular drugs approved by the US Food and Drug Administration from 2008 through 2020. Pivotal trials provide evidence that the Food and Drug Administration uses to approve novel therapeutic agents. Thus, the authors assert, these are trials with direct, immediate clinical impact for which involvement of women investigators is most urgent. The authors analyze 53 trial publications, representing 235 331 participants and 26 novel cardiovascular drugs, and observe the overall proportion of women authors to be just 10%.^5^ Reading between the lines, these results diminish the hope that disparities in trial leadership are narrowing over time in 2 ways. First, Shahid et al^5^ present the proportions of women authors by drug sorted by approval year. Although we are encouraged by the pace of innovation in cardiovascular therapeutics, we are simultaneously disappointed to see that the proportion of women authors has remained stagnant over this time period. Second, the authors report similarly low overall proportions of women first authors (7.5%) and senior authors (11.3%) of publications.^5^ One might expect to observe a larger proportion of women first authors over this 13-year period in a field trending toward equity, but such a pattern is indiscernible here.

Muquith et al^6^ evaluate the recognition of female investigators as authors of trial publications of phase 3 oncology clinical trials. Similar to Shahid et al,^5^ the authors focus on high-impact trials, identifying 114 randomized clinical trials (RCTs) published in the *New England Journal of Medicine, Lancet,* and *Lancet Oncology* in 2019 and 2020.^6^ They characterize disparities in oncology clinical trial publication authorship by exploiting the fact that publications’ authors and steering committee members are typically a subset of a trial’s investigators. They find that while 27.6% of 15 805 investigators were females, female investigators comprised only 22.9% of trial authors and 20.3% of steering committee members.^6^ Like Shahid et al,^5^ they report similar proportions of female first (21.9%) and senior (19.3%) authors among examined publications.^6^ Muquith et al^6^ also leveraged the World Health Organization Global Health Workforce Statistics database to illuminate the association between female physician and female investigator prevalence by country and show that global underrepresentation of female investigators persists across all but 2 of the 52 countries included in their analysis. Their findings allude to the existence of processes that disproportionately exclude women from achieving roles in oncology trial leadership.

Amid a growing body of research that continues to show the underrepresentation of women in academic medicine, it is tempting to become disheartened. Nevertheless, the history and achievement of medicine is the identification of problems and dogged pursuit of solutions for the betterment of humankind. Situating this work within that tradition, we are instead inspired to continue the quest for sex and gender equity in academic medicine. How can it be achieved? Both Shahid et al^5^ and Muquith et al^6^ delineate their findings across disease subtypes. In the analysis by Shahid et al,^5^ pivotal trials of drugs to treat hypercholesterolemia appear to have the highest proportion of women authors, compared with other cardiovascular diseases. Muquith et al^6^ present results in line with previous investigations^7^ showing that certain cancer subtypes, such as breast and gynecological, have a greater proportion of female lead authors compared with oncology clinical trials as a whole. The reasons for these findings cannot be elucidated from the current analyses. However, we can hypothesize that women are more often leaders of trials in hypercholesterolemia as more women cardiologists practice in noninvasive cardiology as opposed to procedural cardiology fields. Similarly, women may lead breast and gynecological cancer trials more often because women investigators may more often study female-specific conditions, a finding that has been demonstrated in the heart failure literature.^4^ Subfields of academic medicine or specific diseases in which inequities in trial leadership are least apparent may provide fruitful fodder as case studies to identify strategies that could be deployed in other fields to reduce sex and gender disparities.

Likewise, Shahid et al^5^ examine the interplay among trial funding, orphan drug status, approval pathway, and proportion of women authors in pivotal cardiovascular therapeutic trials. They find trends toward increased proportion of women authors among trials of therapeutics with orphan drug status and those receiving industry funding from the US as compared with international funders. Ludmir et al^7^ previously demonstrated lower rates of female corresponding authorship in phase 3 oncology trials funded by industry sponsors. Given the substantial role played by industry partners in the clinical trial enterprise, addressing factors specific to market and industry support for female investigators is another opportunity for advocacy to eliminate disparities in academic medicine. Finally, Muquith et al^6^ show that the Philippines and Vietnam are the only 2 countries where the proportion of female investigators is greater than the proportion of female physicians—a reminder that the discovery and implementation of novel therapeutics is a global effort and that we in the US can learn a great deal from our international partners in medicine.

The work of identifying, characterizing, and eliminating sex and gender disparities in academic medicine is arduous but necessary. We are still at its outset, and each benchmarking study, such as these from Shahid et al^5^ and Muquith et al,^6^ illuminates incrementally more of the path forward. As the old saying goes, “a rising tide lifts all boats.” By exploring new avenues to address disparities in clinical trial authorship, we will empower women investigators, strengthen the validity of clinical trial results for female patients, and ultimately achieve a more just and equitable society.
